# Proteomic profiling of circulating extracellular vesicles from COVID-19 patients and their impact on innate Vdelta2 T-cell response

**DOI:** 10.3389/fimmu.2026.1748398

**Published:** 2026-02-11

**Authors:** Claudia Montaldo, Eleonora Cimini, Eleonora Tartaglia, Manuela Antonioli, Veronica Bordoni, Stefania Notari, Michela Notarangelo, Eleonora Torchia, Giulia Canarutto, Silvano Piazza, Vito Giuseppe D’Agostino, Valentina Mazzotta, Luisa Marchioni, Andrea Antinori, Chiara Agrati, Raffaele Strippoli

**Affiliations:** 1 Gene Expression Laboratory, National Institute for Infectious Diseases Lazzaro Spallanzani, IRCCS, Rome, Italy; 2 Cellular Immunology and Pharmacology Laboratory, National Institute for Infectious Diseases Lazzaro Spallanzani, IRCCS, Rome, Italy; 3 Laboratory of Virology and Biosafety Laboratories, National Institute for Infectious Diseases Lazzaro Spallanzani, IRCCS, Rome, Italy; 4 Clinical and Research Infectious Diseases Department, National Institute for Infectious Diseases Lazzaro Spallanzani, IRCCS, Rome, Italy; 5 Department of Biology, University of Rome “Tor Vergata”, Rome, Italy; 6 Department of Hematology/Oncology, Cell and Gene Therapy, Bambino Gesù Children’s Hospital (IRCCS), Rome, Italy; 7 Department of Cellular, Computational and Integrative Biology (CIBIO), University of Trento, Trento, Italy; 8 Computational Biology Unit, International Centre for Genetic Engineering and Biotechnology, ICGEB, Trieste, Italy; 9 Life Science Department (DSV), University of Trieste, Trieste, Italy; 10 Department of Molecular Medicine, Sapienza University of Rome, Rome, Italy

**Keywords:** EVS, extracellular vesicles, gamma delta T lymphocytes, inflammatory cytokines, quantitative proteomics, SARS-CoV2

## Abstract

**Introduction:**

The crosstalk between immune cells through plasma extracellular vesicles (EVs) during SARS-CoV-2 infection may represent a significant determinant of clinical course in COVID-19 patients. EVs from SARS-CoV-2 virus-infected cells deliver their informational content to immune cells implicated in COVID-19 pathogenesis, thereby modulating pro-inflammatory immune responses during infection. γδ T cells are innate cells known for their pleiotropic properties spanning both innate and adaptive immunity and for their possible contribution to inflammation. This study aimed to characterize the biophysical profile and protein content of EVs derived from patients with severe and mild COVID-19, and to analyze their impact on the functional activity of Vδ2 T cells.

**Methods:**

Plasma samples from 42 COVID-19 hospitalized patients (17 severe and 25 mild) were enrolled at the National Institute for Infectious Diseases Lazzaro Spallanzani in Rome. Twenty-three healthy donors (HD) served as the control group. Plasma cytokines were quantified by an automated multiplex immunoassay. EVs were purified using nickel-based isolation (NBI) and analyzed by quantitative LC-MS proteomics. Data are available via ProteomeXchange with identifier PXD072061. Characterization of EVs was performed using multiparametric flow cytometry, as well as the Vδ2 T cell functional assays. Peripheral blood mononuclear cells from 10 HD were utilized for immunological assays.

**Results:**

Cytometric characterization revealed that EVs from severe COVID-19 patients were enriched in platelet components compared to HD and mild patients. Protein expression of EVs from severe patients clustered differently in PCA and heatmap analyses with respect to HD and mild patients. A volcano plot revealed several proteins that were differentially expressed between EVs from mild and severe patients. A significant induction of several processes, including platelet degranulation, complement, coagulation, and innate immunity, was observed in the pathway analysis. EVs from severe COVID-19 patients enhanced the responsiveness of Vδ2 T cells to phosphoantigen, increasing their activation and proinflammatory cytokine production (TNF-α).

**Conclusions:**

Proteomic differential analysis reveals the expression/regulation of innate immune-related proteins in EVs from severe patients compared to mild patients/HD and supports their potential role in modulating innate immunity. Specifically, functional analysis of Vδ2 T cells suggests that EVs may contribute to the pathogenesis of severe COVID-19 by delivering molecular signals that exacerbate innate immune-driven inflammation.

## Introduction

The severe acute respiratory syndrome coronavirus 2 (SARS-CoV-2), a positive-sense, single-stranded RNA virus, is the causative agent of COVID-19 and is responsible for more than 7 million deaths globally since its emergence in 2019 (1, 2). SARS-CoV-2 infects several cell types, with a tropism for various organs, including the respiratory tract, kidneys, liver, heart, brain, and blood vessels (3). COVID-19 patients can be asymptomatic, have mild symptoms such as fever, cough, and dyspnea, or develop into severe conditions, characterized as acute respiratory distress syndrome (ARDS) requiring mechanical ventilation (4). Severe SARS-CoV-2 infection is characterized by a massive host inflammatory response, leading to tissue damage and paralysis of the protective immune response (5–7). The mechanisms underlying the unbalanced immune response are not fully defined, and the molecular signals delivered by extracellular vesicles (EVs) released by infected or stressed cells may play a prominent role.

EVs are nanosized lipid-bilayer vesicles that carry nucleic acid and protein cargo and are widely recognized as mediators of intercellular communication (8). Virtually all types of cells constitutively secrete EVs, circulating in biofluids such as blood, urine, saliva, and breast milk (9). EVs generated from virus-infected cells can act as carriers of virulence factors, including viral proteins and genetic material. Indeed, they contain several biomolecules (such as proteins, lipids and nucleic acids) which are delivered to cell targets, reprogramming their functions and also acting as immunomodulators EVs (10–13).

In COVID-19 disease, EVs have been demonstrated to be implicated in cell-to cell communication between immune and immune-stroma cells during the inflammatory response, impacting on disease severity. Thus, they have attracted growing interest and represent potential diagnostic tools to track disease progression (14–17).

γδ T cells represent about 1-5% of total circulating T cells in the peripheral blood of human adults, and they are also present in various tissues (18, 19). γδ T cells functionally interact with other immune players such as dendritic cells, macrophages, NK cells, and B cells, αβ T lymphocytes, thus acting as a link between the innate and adaptive immunity (20, 21). During the inflammatory/immune response, γδ T cells may exert both pro-inflammatory and anti-inflammatory activities, depending on the immunological context. They can induce inflammation by producing cytokines like IL-17, IFN-γ, and TNF-α, and also promote tissue repair and homeostatic responses. Their effector function is primarily related to their cytolytic potential and to the secretion of immunomodulatory factors (19, 22).

The Vδ2 T cell subset, the major γδ T cell sub-population in humans, is reduced in severe COVID-19 in a frame of a general lymphopenia. In this condition, Vδ2 T cells were found to express both activation and exhaustion markers and to secrete proinflammatory cytokines, including TNFα (5, 23, 24). Among lymphocyte subsets, Vδ2 T cell have been incompletely studied in COVID-19, and in particular, the impact of EVs on their functions has not been analyzed to our knowledge.

In this study, the relative abundance and protein cargo of EVs derived from severe and mild COVID-19 patients, as well as and their immunomodulatory activity on Vδ2 T-cell response, were analyzed. Data from proteomic analysis showed differential expression of EV cargo proteins on the basis of COVID-19 severity. Moreover, functional tests demonstrated that EVs from COVID-19 patients impact on Vδ2 T-cell activation (HLA-DR expression) and function (TNF-α production), suggesting a role of EVs in the γδ T cell-mediated immunopathology of COVID-19.

## Materials and methods

### Study design and cohort

This study was conducted at the National Institute for Infectious Diseases Lazzaro Spallanzani in Rome, Italy. The study was approved by the institutional review board (approval number: 9/2020), and written informed consent was obtained from patients. 17 severe and 25 mild hospitalized COVID-19 patients were selected according to the WHO categorization [https://www.who.int/news-room/fact-sheets/detail/coronavirus-disease-(covid-19)] with a laboratory-confirmed diagnosis of SARS-CoV-2 infection. 23 healthy donors (HD) were enrolled as the control group. Quantitative proteomic analysis and functionality assays was performed on EVs purified from severe (N = 8) and mild COVID-19 (N = 12) patients compared to those of the HD (N = 5) Further details are in the Results section.

### SARS-CoV-2 diagnosis

The following commercial chemiluminescence microparticle antibody assays (CMIA): the SARS-CoV-2 specific anti-N and the anti-S/RBD tests [AdviseDx SARS-CoV-2 IgG II and SARS-CoV-2 IgG II Quant, respectively, ARCHITECT^®^ (Chicago, IL, USA) i2000sr Abbott Diagnostics, Chicago, IL, USA] were used, according to the manufacturer’s instructions. Index > 1.4 and binding antibody units (BAU)/mL>7.1 are considered positive, respectively. Viral RNA was extracted using the QIAsymphony (QIAGEN, Hilden, Germany), and real-time reverse transcription polymerase chain reaction (RT-PCR) targeting the E and RdRp viral genes was employed to assess the presence of SARS-CoV-2 RNA (25). Confirmation of diagnosis was performed by in-house RT-PCR targeting the viral membrane protein (M) gene, followed by Sanger sequencing (327 bp). Follow-up of the infection course was then performed using only the E gene real-time RT-PCR. Other respiratory tract infections were investigated using a multiplex nucleic acid test (QIAstat-Dx Respiratory Panel, QIAGEN, Hilden, Germany).

### Blood sampling

Plasma samples were obtained by centrifuging heparin-anticoagulated peripheral blood for 10 minutes at 1800 rpm, then immediately stored at -80 °C until analysis. Peripheral blood mononuclear cells (PBMCS) from HD were isolated using gradient centrifugation (Lympholyte, Cedarlane, Canada), counted with Trypan blue, and prepared for further experiments.

### Cytokine detection

Inflammatory cytokines (IL-8, Il-1β, IL-6, TNF-α) in plasma samples were quantified using an automated multiplex immunoassay on the Ella instrument (Protein Simple/Biotechne, San Jose, CA, USA). Ella is an advanced benchtop automated ELISA system created to provide precise, consistent data without requiring any manual procedures. The Ella platform’s Simple Plex™ assays, featuring a distinctive microfluidic design, are fully validated, extremely sensitive, and capable of measuring up to 8 analytes at once. The detection limit is 0.19 pg/mL for IL-8; IL-1β: 0.064 pg/mL; IL-6: 0.11 pg/mL; TNF-α: 0.03 pg/mL.

### EVs enrichment and characterization

A rapid nickel-based approach was used to isolate plasma EVs. In particular, this method exploits nickel cations’ capacity to capture EVs and uses chelating agents to elute non-aggregated vesicles in a physiological pH solution, preserving their integrity and functional capacities (26). Briefly, 1 ml of plasma sample was centrifuged at 2800 g for 10’ to remove potential cellular contaminants and diluted 1:3 in PBS 1X. After 30’ incubation with nickel beads in shaking at RT, the EVs were eluted in a suitable elution volume by shaking for 10’ at 28 °C. The EV characterization in terms of size and concentration was performed using Nanoparticle Tracking Analysis (Nanosight). The results were analyzed using NanoSight NTA3.4 software.

### EVs flow cytometry

Phenotype and frequency of EVs were evaluated by multiparametric flow cytometry using the Dye Integer EV Detection kit (Becton-Dickinson, San Jose, USA), which contains a lipophilic cationic dye that diffuses into EVs based on their membrane potential, and FITC-conjugated phalloidin, which binds to the cytoskeletal protein actin in vesicles with damaged membranes. Absolute cell counts were obtained using a TruCount absolute counting tube (Becton-Dickinson, San Jose, USA). The staining mix was prepared in PBS 1X (Corning) by first adding the lipophilic cationic dye and phalloidin, followed by the antibody cocktail consisting of anti-CD45 BV510, anti-CD41a PE, and anti-CD31 PE-Cy7. The antibodies and fluorochromes used are described in **Supplementary Figure 1**. Before use, the staining mix was centrifuged at 16,000x g for 15 minutes to remove particles. Samples’ plasma was processed according to the kit’s procedure. Briefly, 50 µl of filtered PBS 1X (Corning) was loaded into a trucount tube without perturbing the bead pellet. After, 50 µl of plasma was added using reverse pipetting and 95 µl of staining mix. After 45 minutes of incubation in the dark at room temperature, the tubes were supplemented with 1 mL of filtered 1X PBS, and the cells were acquired using a BD FACSLyric (Becton-Dickinson, San Jose, USA). The cells were then analyzed using BD FACSuite (Becton-Dickinson, San Jose, USA). Gating Strategy Analysis: A dot plot was used to display phalloidin, and a lipophilic cationic dye (CLP) was generated to create a gate in the phalloidin-negative area, thereby excluding vesicles with damaged membranes (**Supplementary Figure 2**). This gate was then applied to a CLP/FSC dot plot to discriminate EV events from the background. The enriched EV area was identified as phalloidin-negative, CLP-positive events. This EV area was further analyzed on a CD45/CD41 dot plot, where CD45+ events were considered leukocyte-derived EVs. A gate excluding CD45+ events was subsequently plotted on a CD31/CD41 dot plot to assess other EV phenotypic populations. CD31+/CD41+ events were identified as platelet-derived EVs, while CD31+/CD41- events were classified as endothelium-derived EVs.

### EVs proteomics and data analysis

For quantitative mass spectrometry analysis, 2,29 E + 09 particles (NTA-determined) per sample were used. The purified EVs were lysed using RIPA buffer and analyzed by a Q Exactive Plus Orbitrap LC-MS/MS system, as described in (27). Briefly, after overnight trypsin digestion (0.6 μg/sample) at 37 °C, the peptide mixture was acidified with 0.5% trifluoroacetic acid and fractionated using the High pH Reversed-Phase Peptide Fractionation Kit (Thermo Fisher Scientific) according to the manufacturer’s protocol. Peptides in each fraction (8/sample) in 2.5% acetonitrile, 0.1% TFA, and 0.1% formic acid solution were then analyzed by a UltiMate 3000 RSLCnano-LC system (Thermo Fisher Scientific) connected online via a nano-ESI source to a Q Exactive plus™ Hybrid Quadrupole-Orbitrap™ Mass Spectrometer (Thermo Fisher Scientific). Each sample was run twice as a technical replicate. Proteins were automatically identified using MaxQuant (v. 1.6.17.0). Tandem mass spectra were searched against the *Homo sapiens* dataset of UniprotKB database. Quantitative comparisons among different samples were performed using the label-free quantification (LFQ) algorithm calculated by MaxQuant. Perseus software (version 1.6.15.0) (28) was used to analyze LC–MS data. Protein intensities obtained from MaxQuant LFQ values were log2-transformed and grouped to compare healthy donors (HD) with COVID-19 patients, stratified by disease severity into mild and severe. Proteins quantified in at least one group with ≥70% coverage were retained, and missing values were imputed using a normal distribution (width = 0.3, downshift = 1.3). The complete data matrix was subjected to Principal Component Analysis (PCA), and the first two components were extracted (FDR < 0.05). For volcano plots, 250 randomized permutation tests based on a two-sided t-test (FDR < 0.05, S0 = 0.1) were applied to identify differentially abundant proteins (DAPs) between mild and severe COVID-19. Hierarchical clustering was performed after Z-score normalization, followed by ANOVA and Tukey’s HSD *post hoc* tests (FDR < 0.05). Clusters were generated using Euclidean distance, yielding 12 clusters across HD, mild, and severe groups. DAPs between mild and severe cases were further analyzed for pathway enrichment using the STRING app in Cytoscape, querying Reactome, KEGG, and WikiPathways databases (FDR < 0.05, redundancy = 0.4). Enrichment results were visualized as a bubble plot in GraphPad Prism 9.0, where bubble color reflected FDR values and bubble size indicated the number of DAPs associated with each pathway term. 

### Functionality tests

To assess the effect of the EVs obtained from sera of COVID-19 patients and HD on the functionality of Vδ2 T cells, we stimulated PBMCs from 11 HD with a Vδ2-specific antigen (phosphoantigen, PhAg, IPH1101, 3 µM, Innate Pharma, France) for 18–20 hours at 37 °C and 0.05% of CO_2_. The resting or PhAg-stimulated PBMC were then treated with EVs from COVID-19 patients and HD [1.7 × 10^9 EVs on 150,000 T γδ (1.14 × 10^9 EVs/cell)] for 18–20 hours. After 1 hour, Brefeldin A (Serva, Euroclone, Italy) was added at 10 µg/mL to block cytokine secretion. At the end of stimulation, PBMCs were stained with a cocktail of monoclonal antibodies: Vd2-FITC, CD3-Percp-Cy5.5, and HLA-DR-ECD. Briefly, PBMCs were stained with mAbs for 20 minutes at 4 °C, then washed with PBS once and fixed with 1% paraformaldehyde (Sigma, Italy). After the fixation, cells were washed and stained for TNF-α (PE) for 15 minutes at RT. Cells were washed and acquired on the DXFlex cytometer (Beckman Coulter). The gating strategy is described in **Supplementary Figure 3**. Data were analyzed with DxFlex software 2.0.2.18 (Beckman Coulter).

### Statistical analysis

The non-parametric Mann-Whitney test was used in this study. The statistical analyses were performed using the GraphPad Prism software 8.0. For proteomic data, all the statistical analyses were performed using Perseus software as described above.

## Results

### Flow cytometric analysis of plasmatic EVs from severe and mild COVID-19 patients

In this study, 17 severe and 25 mild COVID-19 patients hospitalized at the National Institute for Infectious Diseases Lazzaro Spallanzani with a laboratory-confirmed diagnosis of SARS-CoV-2 infection were selected, according to the WHO categorization. The 76.4% (13/17) of severe COVID-19 subjects were hospitalized in the Intensive Care Unit (ICU). Among the cohort of severe subjects, 8 ICU hospitalized-subjects died. 23 healthy donors (HD) were enrolled as the control group. **Table 1** describes the clinical and biological features of the study cohort, showing age media, gender, hospitalization status, and mortality.

**Table 1:** Clinical and hematological parameters of the enrolled patients (plasma cytokine detection, cellular origin of EVs).

Patients	Mild	Severe	P value
Age media (range)	63 (46-95)	66 (33-81)	0.311
Gender (F/M)	5/20	4/13	Not applicable
Hospitalization (%)	68% (17/25)	100% (17)	Not applicable
Mortality (%)	0%	47% (8/17)	Not applicable
Leukocytes Count (x10 e3/uL)	5.7 (4.2-8.2)	8.9 (5.8-12.0)	0.03
PCR (mg/dL)	4.1 (1.6-13.0)	16.7 (10.4-21.7)	0.001
D-Dimer (ng/mL)	710 (401- 4234)	958 (559-3982)	0.433
Fibrinogen (mg/dL)	650 (465-824)	618 (584.5-944)	0.488
% CD3 Median (IQR)	73.4 (67.1- 76.8)	61.9 (55.9-68.1)	0.01
% CD4 Median (IQR)	41.1 (32.1- 50.8)	49.1 (39.4-54.8)	0.203
% CD8 Median (IQR)	22.2 (15.4-32)	13.5 (7.6-17.3)	0.0003
% NK Median (IQR)	20.4 (5.2-20.5)	13.7 (10.9-16.9)	0.007
% B Median (IQR)	4.5 (3.1-9.8)	23.5 (14.7-27.6)	0.692
IL-6 (pg/mL) Median (IQR)	5 (2.5-26)	77.5 (22.4-288.2)	0.0003

**Figure 1:**
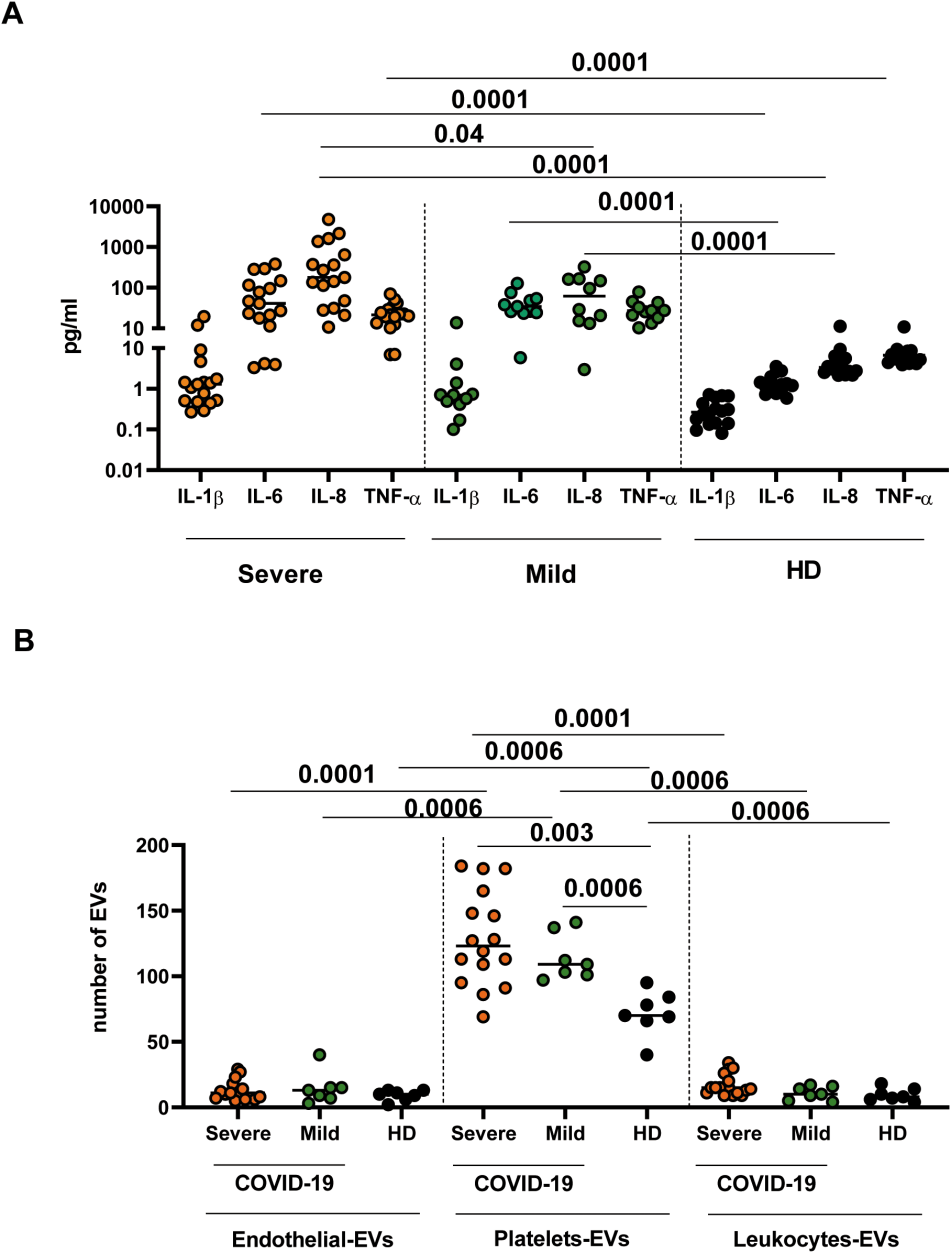
Inflammatory cytokines analysis and cytometric EVs characterization from mild and severe subjects and from healthy controls. **(A)** Inflammatory cytokines (IL-1β, IL-6, Il-8 and TNF-α) were tested in plasma of 17 mild, 11 severe COVID-19 subjects and 16 HD with an automatic multiplex immunoassay. **(B)** EVs from ten mild, 8 severe COVID-19 subjects, and from 7 HD were analyzed by multiparametric flow cytometry. Values were reported as medians, and p-values < 0.05 were considered significant.

The sample cohorts enrolled in this study were first analyzed for plasmatic inflammatory cytokine detection by ELISA. As expected, severe patients showed higher levels of pro-inflammatory cytokines [IL-6 (p<0.0001), IL-8 (p<0.0001), and TNF-α (p<0.0001)] with respect to both HD and mild patients [IL-6 (p<0.0001), IL-8 (p<0.0001)]. (**Figure 1A**). EVs extracted from plasma samples of severe and mild patients, as well as of HD, were isolated through a nickel-based approach. To analyze the cellular origin of the EVs, we compared the expression of key markers of platelets (CD31+/CD41+), leukocytes (CD45+/CD41-), and endothelium (CD31+CD41-) among severe, mild COVID-19, and HD. Data in **Figure 1B**, show a higher frequency of platelet-derived EVs in severe subjects than in HD (p<0.0006) and in mild subjects than in HD (p<0.003). No differences were observed between endothelial and leukocyte EVs. EVs among the three groups. Additionally, both severe and mild COVID-19 patients showed a higher number of EVs-platelet-derived compared to the EVs-endothelial-derived (severe p<0.0001; mild p<0.0006) and to the Leukocytes-derived EVs (severe p<0.0001 and mild p<0.0006).

### Proteomic analysis of plasmatic EVs purified from severe and mild COVID-19 patients

To characterize EVs in term of nanoparticle size, concentration and proteomic cargo, a nanoparticle tracking analysis following by quantitative proteomic analysis was performed on EVs purified from severe (N = 8) and mild COVID-19 (N = 12) patients compared to those of the HD (N = 5). The clinical and biological features of this sub-cohort are reported in **Table 2**.

**Table 2:** Clinical and hematological parameters of the enrolled patients (EVs undergoing quantitative proteomics and used for functional tests).

Patients	Mild	Severe	P value
Age media (range)	53 (46-27)	66 (60-76)	0.18
Gender (F/M)	5/17	2/17	Not applicable
Hospitalization (%)	70.5% (12/17)	100% (8/17)	Not applicable
Mortality (%)	0% (0/12)	47% (8/17)	Not applicable
Leukocytes Count (x10 e3/uL)	4.3(4.0-8.3)	10.7 (5.0-13.5)	0.04
PCR (mg/dL)	1.6 (0.05-11,7)	6.1 (4.1-28.2)	0.06
D-Dimer (ng/mL)	511 (167-1513)	1518 (622-3637)	0.25
Fibrinogen (mg/dL)	416 (231-824)	671.5 (416-1111)	0.07
% CD3 Median (IQR)	74.6 (44.2-83.2)	57.8 (43-79)	0.38
% CD4 Median (IQR)	49.1 (37.9-61.5)	50 (23.4-65.3)	0.83
% CD8 Median (IQR)	21.9 (11.3-34,1)	8.7 (4-17.3)	0.11
% NK Median (IQR)	Not performed	27.4 (12.5-28.1)	Not applicable
% B Median (IQR)	Not performed	14.9 (8.4-20.0)	Not applicable

**Figure 2:**
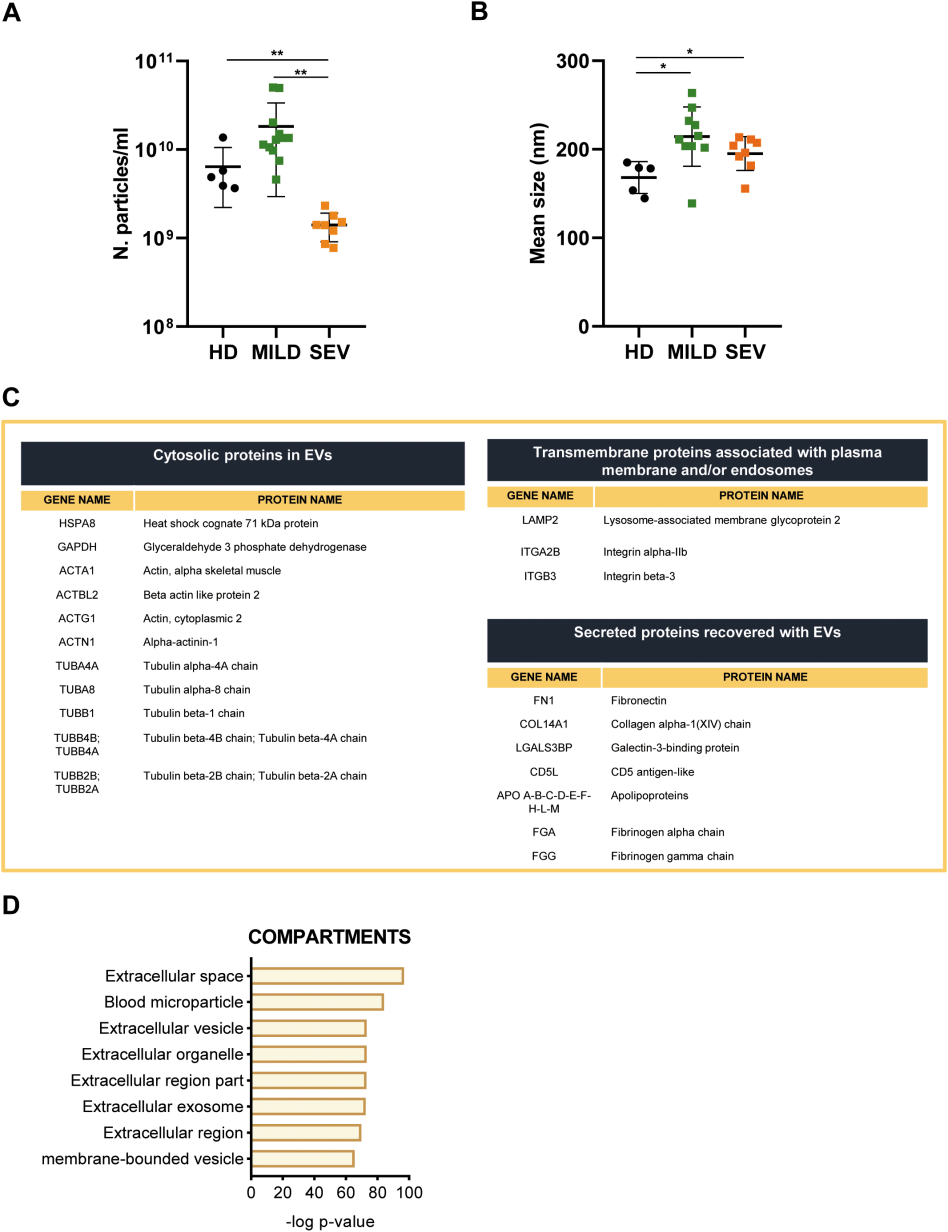
EVs characterization from mild and severe subjects and from healthy controls. **(A, B)** EVs concentration (n. particle/ml) and mean size (nm) detected by NTA. Differences were considered significant *p < 0.05; **p < 0.01. **(C)** Table showing identified EVs protein in the entire dataset analyzed by the proteomic approach and listed as suggested by MISEV 2023 **(D)** Proteomic data were analyzed using Enrichr with the Jensen_COMPARTMENTS subcellular localization database, considering the entire dataset obtained from EVs isolated from the plasma of healthy donors (HD) and COVID-19 patients.

As shown in **Figure 2A**, the number of nanoparticles in severe COVID-19 samples was significantly lower compared to HD and mild COVID-19 EV samples; on the other hand, nanoparticle mean size was higher in mild and severe COVID-19 samples with respect to HD (**Figure 2B**).

**Figure 3:**
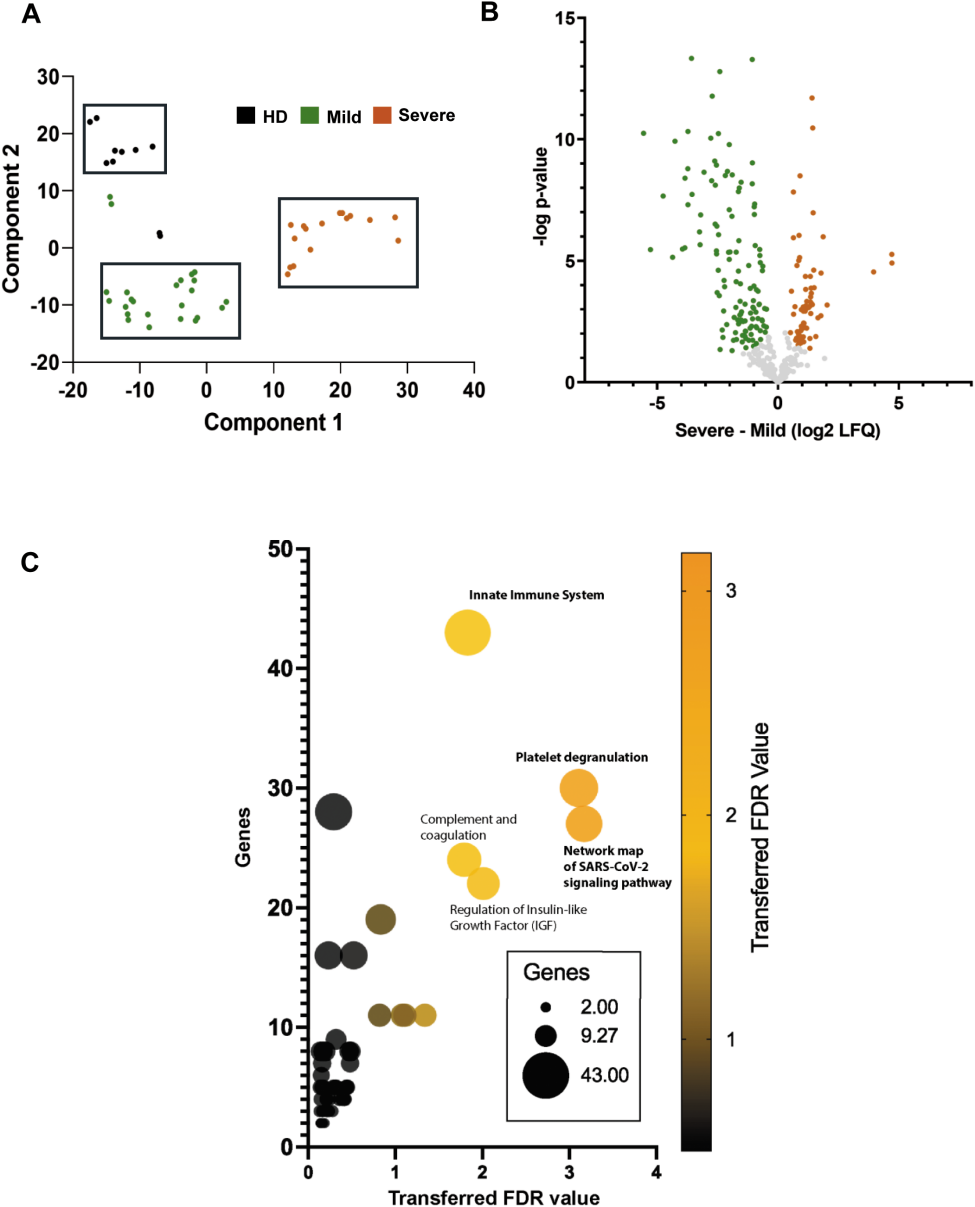
Proteomic analysis of EVs from HD and mild/severe COVID-19 patients **(A)** Principal component analysis (PCA) of EVs derived from the plasma of HD and COVID-19 patients, stratified according to disease severity (mild, severe). **(B)** Volcano plot illustrating the differentially expressed proteins between mild and severe COVID-19 cases. **(C)** Bubble plot summarizing the most significantly enriched pathways modulated between mild and severe patients based on the volcano plot analysis. The color gradient reflects the adjusted false discovery rate (FDR), while bubble size represents the number of proteins associated with the same KEGG, Reactome, or Wikipathways terms.

A total of 348 proteins were identified by MaxQuant software (**Supplementary Table 1**). Among them, in accordance with the “minimal experimental requirements for definition of EVs and their functions,” several proteins that properly assess EVs’ features were identified (29). In particular, we identified EVs transmembrane proteins associated with the plasma membrane and/or endosomes (LAMP2, ITGA2B), cytosolic EVs proteins (HSPA8, GAPDH), and secreted proteins recovered with EVs also or “corona “proteins (FN1, COL14A1, LGALS3B) (**Figure 2C**). Furthermore, also gene ontology analysis revealed that the most enriched cellular compartments in EVs extracts analyzed are extracellular space, blood microparticle and extracellular vesicle, as expected (**Figure 2D**). Next, to identify differentially abundant proteins (DAPs) across three sample datasets, a computational analysis was conducted. Principal component analysis (PCA) revealed three separated cluster groups corresponding to healthy donors, mild, and severe COVID-19 samples (**Figure 3A**). To highlight statistically significant DAPs between severe and mild COVID-19 EVs samples, a Volcano plot analysis was applied. In particular, 68 DAPs were increased and 125 DAPs decreased in severe COVID-19 EVs samples with respect to mild COVID-19 EVs (**Figure 3B**; **Supplementary Table 2**). Among them several proteins involved in complement activation (C4, C5, C6, C9), coagulation cascades (SERPINF2, FN1) and SARS-CoV-2 signaling pathway (SAA1, SAA2, CRP, FGA) were identified. Indeed, bubble plot analysis based on DAPs detected by Volcano plot reveals Innate Immune System, platelet degranulation and network map of SARS-COV-2 signaling pathway as the most significant enriched pathways modulated between mild and severe patients (**Figure 3C**). All the DAPs belonging to each enriched pathway are listed in **Supplementary Table 3**. Taken together, these proteomic data provide evidence that circulating EVs derived from severe and mild COVID-19 patients differ in protein composition and their specific cargoes, suggesting a role for EVs in innate immune system regulation during COVID-19 disease.

### Immunomodulatory activity of EVs on Vδ2 T-cell functionality

Given that the innate immune system emerged as one of the most significantly enriched pathways in severe COVID-19, we focused on Vδ2 T cells, a subpopulation able to produce large amounts of pro-inflammatory mediators. To investigate the immunomodulatory effect of EVs obtained from COVID-19 patients on the Vδ2 T cells response, PhAg-stimulated PBMC from HD were incubated with EVs derived from severe (n=8) and mild (n=10) COVID-19 subjects, and from healthy controls (n=5) also subjected to proteomic analysis. PhAg-stimulated PBMCs without EVs were used as a control.

**Figure 4:**
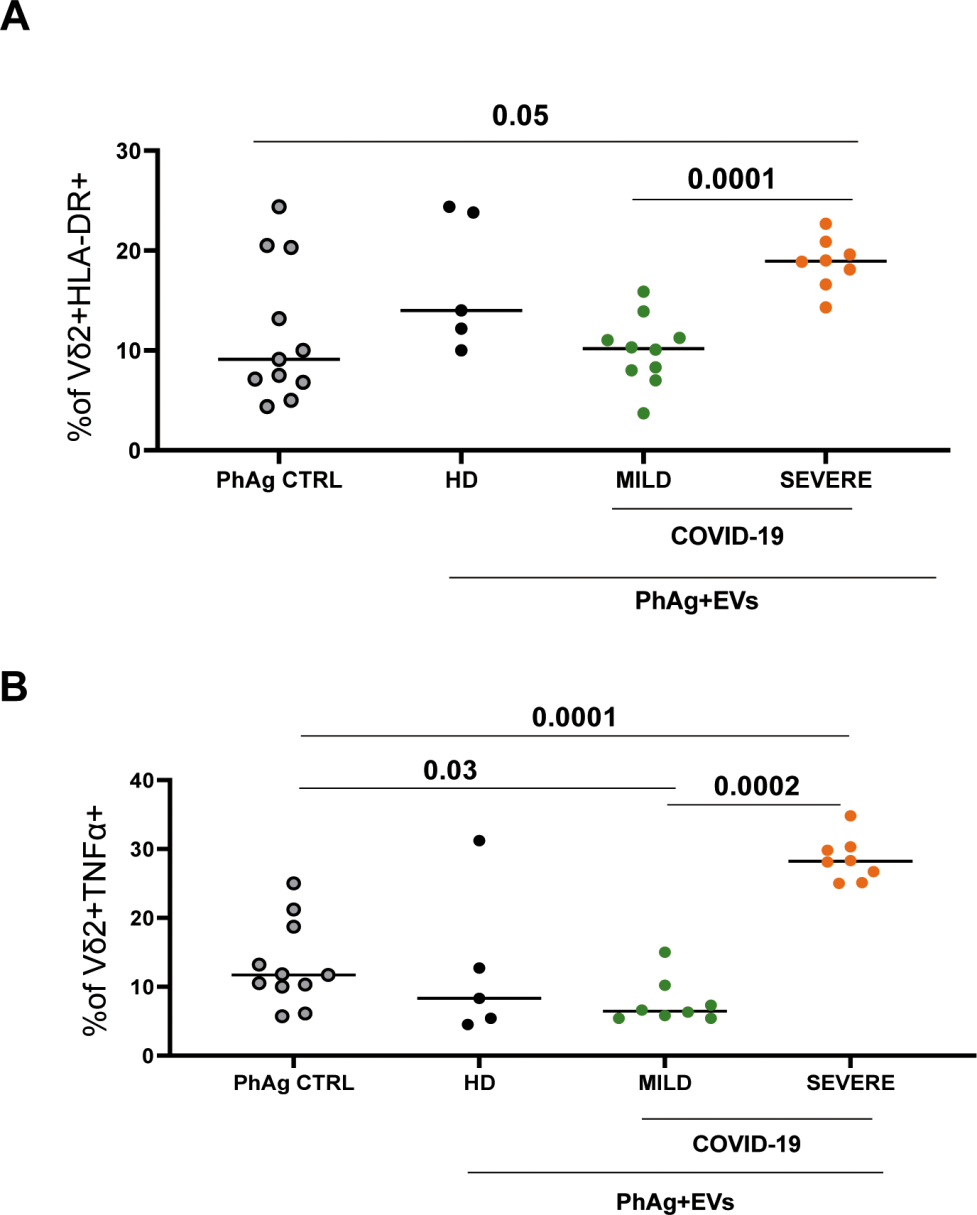
Vδ2 T-cells cytokine production and HLA-DR expression. **(A)** HLA-DR expression and **(B)** TNF-α production by Vδ2 T cells from 11 HD after PhAg stimulation and EVs treatment were shown. Specifically, PBMC from 11 HD were stimulated with PhAg and treated with EVs from 8 severe, 10 mild, and 5 HD subjects for 18–20 hours. Brefeldin A was added after one hour of incubation. Cells were acquired and analyzed by multiparametric flow cytometry. Data were shown as median values. Values <0.05 were considered significant.

At first, we analyzed the modulation of the HLA-DR on Vδ2 T cells. As shown in **Figure 4A**, EVs from severe COVID-19 patients induced a higher frequency of Vδ2 T cells expressing HLA-DR compared to EVs from mild (p<0.0001) or EVs-untreated control (p<0.05). Moreover, we analyzed the effect of EVs on Vδ2 T cell functionality. As shown in **Figures 4A, B**, EVs from severe COVID-19 patients induced a higher frequency of TNF-α-producing Vδ2 T cells compared with EVs from mild patients (p < 0.0002) and with EV-untreated cultures (p < 0.0001). Moreover, the treatment with EVs from mild patients induced a significant decrease compared EVs-untreated control (p<0.03). In contrast, no significant effects of EVs derived from the sera of HD were observed.

## Discussion

The aim of this study was to analyze the structural and informational content of EVs of severe and mild COVID-19 patients to further elucidate pathways leading to severe COVID-19 pathogenesis. Several studies have highlighted the pivotal role EVs in the pathogenesis of SARS-CoV-2 (14–16, 30). Infected host cells secrete EVs containing viral RNA, proteins, and various bioactive molecules, which may facilitate viral spread and influence host immune responses (1). Additionally, EVs have potential as noninvasive diagnostic biomarkers and as cargoes for the therapeutic delivery of biomolecules or drugs (12). Notably, SARS-CoV-2 infection alters the features of serum-derived EVs (31).

In our study, the analysis of plasma samples confirmed elevated levels of proinflammatory cytokines (IL-6, IL-8, and TNF-α), underscoring the inflammatory milieu in severe COVID-19 (30). Moreover, the presence of a higher level of platelet-derived EVs highlighted a significant impact of severe COVID-19 on the coagulation pathway, as previously described (17, 32). Notably, specific cargo of circulating EVs is considered a rich source of information in SARS-CoV-2 pathogenesis and progression that could be highlighted by innovative omics technologies. According to literature, EVs quantitative proteomic analysis reveals significant differences of several proteins involved in the innate immune system and platelet degranulation, which are the main mechanisms of COVID-19–associated tissue damage and multiple organ dysfunctions (33). The observed increased expression of C5 and C9 complement proteins in severe COVID-19 patients is in accordance with previous studies reporting the activation of the complement system in severe COVID-19. These studies provide a rationale for COVID-19 treatment using the C5 inhibitory monoclonal antibody Eculizumab (34).

Moreover, as expected during SARS-CoV-2 infection, proteins linked to inflammatory response and tissue damage such as Serum amyloid A-1 protein A-2 and C-reactive protein (CRP), were found strongly upregulated in EV proteome of severe compared to mild COVID-19 patients. With respect to CRP, it has been demonstrated that during sepsis it is associated to EVs in a monomeric rather than pentameric form, gaining pro-inflammatory properties (35). However, due to the methodology of EV isolation, we cannot rule-out that at least a part of these proteins derive from plasma contamination rather than genuine EV-associated cargo.

Enriched pathways analysis revealed that innate immune system, platelet degranulation and network map of SARS-COV-2 signaling pathway were among the most significant enriched pathways differentially expressed. Among the downregulated proteins, several proteins are implicated in complement activation and coagulation. Their decreased expression may reflect increased consumption secondary to massive activation of these pathways. In detail, mannan-binding lectin serine protease (MASP) 1 and 2 have been linked to complement activation by SARS-CoV2, and MASP-2 abundance correlated with complement activation and inflammatory markers in COVID-19 patients (36, 37). Mannose-binding protein C (MBC) is implicated in both complement induction and γδ T cell activation (38). Lower levels of complement negative regulatory factor C4-BP in severe COVID-19 patients may be related to SARS-CoV2 ability in suppressing complement negative regulation during critical COVID-19 disease (39).

Moreover, proteins related to extracellular matrix protein (ECM) and TGFβ activation were found, such as vitronectin, fibronectin and SPARC. These molecules are relevant for γδ T cell adhesion to ECM and activation (40). Downregulation of TGFβ signature may have a role in the altered γδ T cell expansion and activation occurring in COVID-19 patients.

Interestingly, proteins linked to glucose regulation and adipogenesis were also strongly downregulated. Adiponectin, previously found in EVs, was demonstrated to suppress the activity of several lymphocytic subpopulations, including γδ T cells. (41, 42). Also, Insulin-like growth factor-binding protein complex acid labile subunit (IGFALS) and Insulin-like growth factor-binding protein 3 (IGFBP3) have been implicated in lymphoid compartment reconstruction and homeostasis, which are defective in SARS-CoV2 patients (43).

Thus, while several proteins were found potentially able to modulate γδ T cell expansion and functionality, further investigation is needed for their validation and provide mechanistic links.

It is well known that EVs may carry various cytokines and chemokines, either on cell surface, bound to glycosaminoglycans, or as internal cargo (11). Specifically, EVs have been demonstrated to carry cytokines potentially impacting on Vδ2 T cells activity such as TNFα, Interferons and TGFβ (44–46). Cytokines and chemokines were not found in the proteomic analysis of our cohort, but we cannot exclude that these extracellular messengers play a role in the observed modulation of Vδ2 T cells activation. Alternatively, several membrane-bound, cargo proteins and noncoding RNAs may play a role in the functionality of recipient immune and parenchymal/stromal cells, as demonstrated by us and other laboratories in other experimental systems (13, 27).

γδ T cells play an essential role in antiviral protection against several emerging viruses (47). They can recognize PhAgs, which are overexpressed in transformed and infected cells (48, 49). An effective activation of Vd2 T cells during SARS-CoV infection has been demonstrated (50). Nevertheless, during severe COVID-19, γδ T cells were reduced (in a frame of a general lymphopenia) and expressed exhaustion markers (5, 23), suggesting that they may participate in the response to SARS-CoV2 infection.

The effect of EVs from COVID-19 patients on Vδ2 T cell activity has not been reported so far to our knowledge. Here, treatment of Vδ2 T cells from HD patients with EVs from severe patients resulted in higher production of TNFα, compared to mild patients and HD, underscoring their possible involvement in the severe phase of SARS-CoV-2 infection contributes to the inflammatory status of severe infection (5, 23). Additionally, we observed higher HLA-DR expression on Vδ2 T cells treated with EVs from severe patients.

Limitations of this study include the small sample size, the single-center design, and potential technical limitations of EV isolation and proteomics due to the limited amount of sample available. Moreover, the precise mechanism of Vδ2 T cells activation by EVs was not elucidated. However, we would like to point out that since patients were recruited before COVID-19 vaccines were accessible in Italy, these results are valuable since they could not be influenced by vaccine immunization. Moreover, despite the large volume of existing literature on proteomics from COVID-19 patients and on the role of immune cells, studies on Vδ2 T cells are relatively limited, and no study to our knowledge has dealt on the functional impact of EVs from COVID-19 patients on these cells.

Overall, our results suggest a possible proinflammatory role for γδ T cells in severe COVID-19, given their unique ability to connect innate immunity with adaptive immune elements, and provide a possible mechanism of intercellular communications via EVs.

## Data Availability

The mass spectrometry proteomics data have been deposited to the ProteomeXchange Consortium via the PRIDE partner repository with the dataset identifier PXD072061 (51).
