# Supplementary material for: Proteomic profiling of circulating extracellular vesicles from COVID-19 patients and their impact on innate Vdelta2 T-cell response
Source: Front Immunol. 2026 Feb 11;17:1748398. doi: 10.3389/fimmu.2026.1748398 (PMC12933268; doi:10.3389/fimmu.2026.1748398)
Supplement: Supplementary file 1 [file DataSheet1.docx]

**Supplementary Material**

**Proteomic profiling of circulating extracellular vesicles from COVID-19 patients and their impact on innate Vdelta2 T-cell response.**

Claudia Montaldo^1*^, Eleonora Cimini^1*^, Eleonora Tartaglia^1^, Manuela Antonioli^1^,^2^, Veronica Bordoni^3^, Stefania Notari^1^, Michela Notarangelo^4^, Eleonora Torchia^4^, Giulia Canarutto^5,6^, Silvano Piazza^5,6^, Vito Giuseppe d’Agostino^4^, Valentina Mazzotta^1^, Luisa Marchioni^1^, Andrea Antinori^1^, Chiara Agrati^3,8^, Raffaele Strippoli^1,7,8^

**Supplementary Figure 1. Antibodies and fluorochromes used for the study are described.**

#
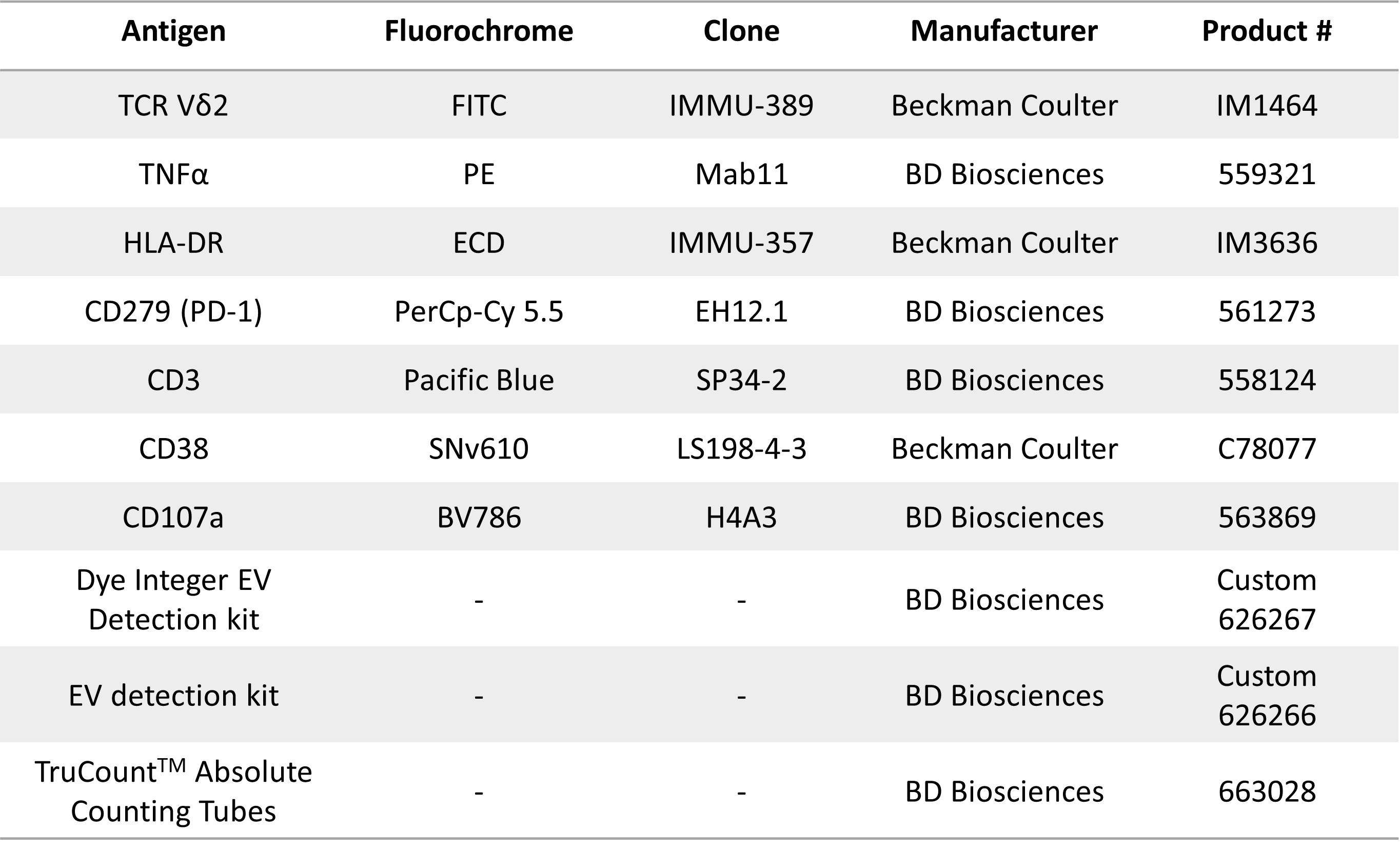


**Supplementary Figure 2. Gating strategy for EVs flow cytometric analysis.**

**
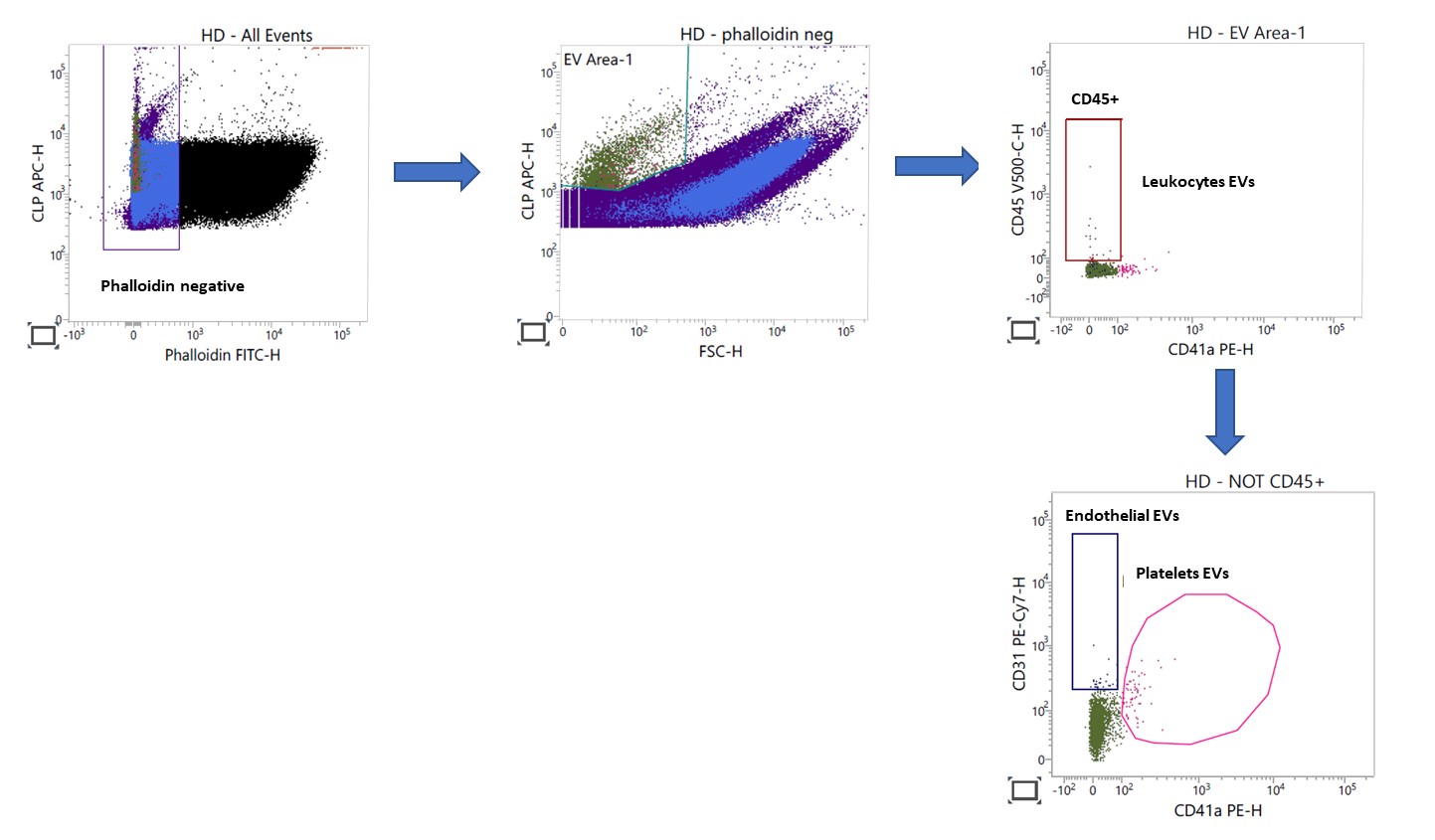
**

**Supplementary Figure 3. Gating strategy for flow cytometry functional test.**

**
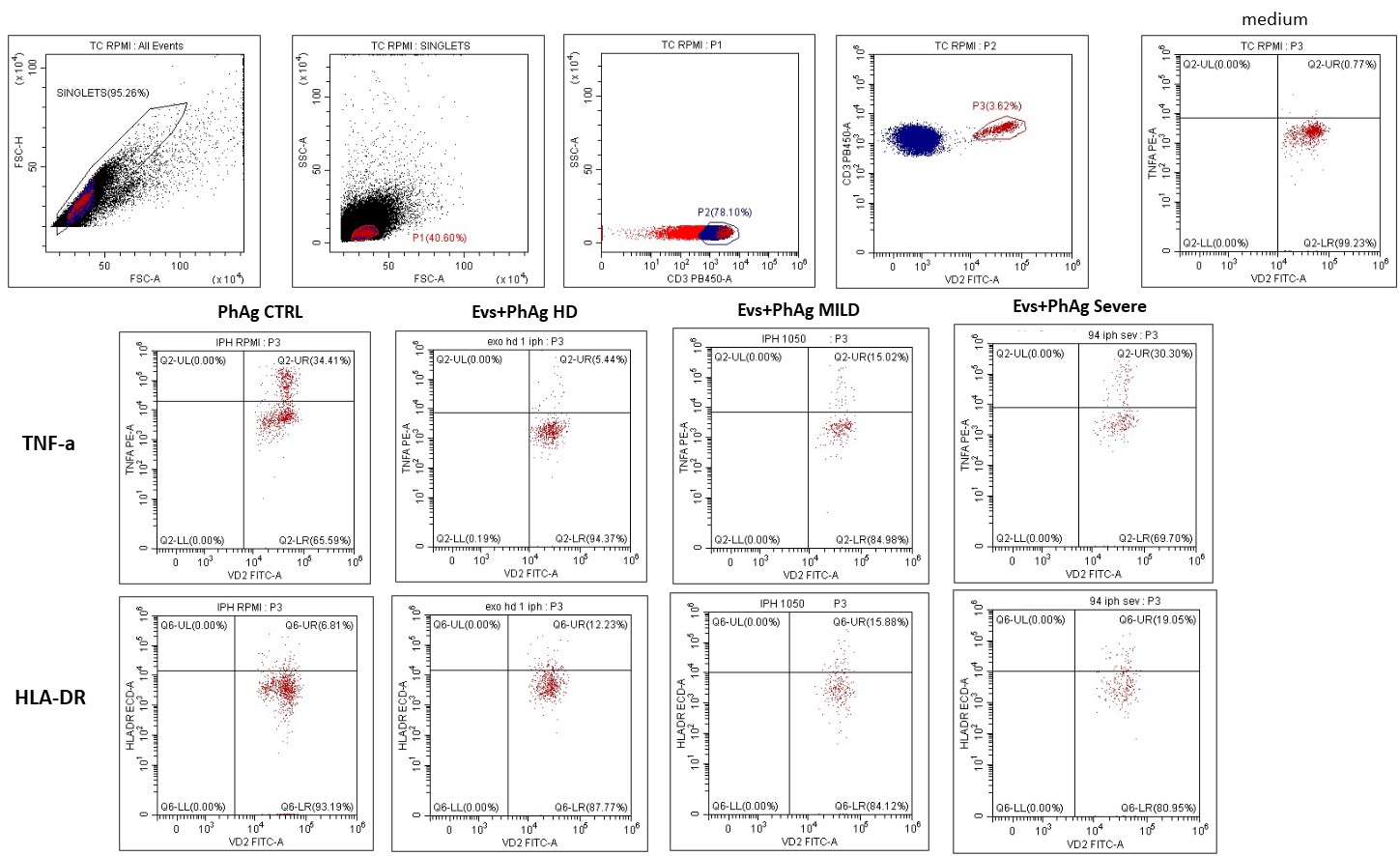
**

## Supplementary Table 1. List of all the proteins identified in the entire dataset of EVs samples analyzed by MaxQuant-Perseus software platform.

## Supplementary Table 2. List of significant statistical and differentially expressed proteins between HD, mild and severe COVID-19 patients highlighted by volcano plot analysis (MaxQuant-Perseus software)

## Supplementary Table 3. List of the most significantly enriched pathways modulated between mild and severe patients based on the volcano plot analysis visualized by bubble plot. (MaxQuant-Perseus software)
